# A Comparison of the Prevalence and Genotype Distribution of Human Papillomavirus Infection Among HIV-Positive and HIV-Negative Women in Lagos University Teaching Hospital, Nigeria

**DOI:** 10.7759/cureus.108295

**Published:** 2026-05-05

**Authors:** Adebola A Adejimi, Kehinde S Okunade, Aderinsola F Faturoti, Kofoworola A Odeyemi, Adekunbiola A Banjo, Alani S Akanmu

**Affiliations:** 1 Department of Community Health and Primary Care, Lagos University Teaching Hospital, Lagos, NGA; 2 Department of Community Health and Primary Care, College of Medicine, University of Lagos, Lagos, NGA; 3 Department of Obstetrics and Gynaecology, Lagos University Teaching Hospital, Lagos, NGA; 4 Department of Obstetrics and Gynaecology, College of Medicine, University of Lagos, Lagos, NGA; 5 Department of Community Medicine, Babcock University Teaching Hospital, Ilishan-Remo, NGA; 6 Department of Anatomic and Molecular Pathology, College of Medicine, University of Lagos, Lagos, NGA; 7 Department of Haematology and Blood Transfusion, College of Medicine, University of Lagos, Lagos, NGA

**Keywords:** cervical cancer, genotype distribution, hiv, human papillomavirus, lagos nigeria

## Abstract

Background: Human papillomavirus (HPV) infection is a major etiological factor in cervical cancer, with increased burden among women living with human immunodeficiency virus (HIV). This study compared the prevalence and genotype distribution of HPV infection among HIV-positive and HIV-negative women in Lagos University Teaching Hospital (LUTH), Lagos, Nigeria.

Methods: A comparative cross-sectional study was conducted among 441 asymptomatic women (232 HIV-positive women attending the AIDS Prevention Initiative in Nigeria clinic and 209 HIV-negative women attending the General Outpatient Department clinic) selected using systematic random sampling. HPV DNA testing was performed to detect the presence and genotypes of HPV. Data were collected using interviewer-administered questionnaires and analyzed using chi-square or Fisher’s exact tests, with statistical significance set at p < 0.05.

Results: The mean age ± standard deviation (SD) of the respondents was 42.47 ± 8.05 years. The overall prevalence of HPV infection was 38.8%. HIV-positive women had a significantly higher prevalence of HPV infection (50.4%) compared to HIV-negative women (25.8%) (p < 0.001). High-risk HPV genotypes were more prevalent among HIV-positive women (91.5%) than HIV-negative women (68.5%) (p < 0.001). HPV 52 was the most common genotype overall. Multiple HPV infections were more frequent among HIV-positive women, though not statistically significant. Several high-risk genotypes, including HPV 18, 45, 52, 53, 58, and 68, were significantly more prevalent among HIV-positive women.

Conclusion: HIV-positive women bear a significantly higher burden of HPV infection, particularly high-risk genotypes. The predominance of non-HPV 16/18 genotypes highlights the need for broader vaccine coverage and strengthened cervical cancer prevention strategies.

## Introduction

Cervical cancer remains a major public health challenge globally, ranking as the fourth most common cancer among women [1]. It was estimated that there were 662,044 new cervical cancer cases and 348,709 deaths of women (particularly middle-aged women) from cervical cancer, corresponding to the fourth cause of cancer morbidity and mortality in women globally in 2022 [1]. Cervical cancer is the second most common female cancer in women aged 15 to 44 years worldwide [1]. Persistent infection with high-risk types of human papillomavirus is the primary cause of cervical cancer, accounting for nearly all cases worldwide [2].

The distribution of cervical cancer differs across the world. The burden of cervical cancer is disproportionately higher in low- and middle-income countries, particularly in sub-Saharan Africa, where access to screening and vaccination remains limited [3]. Nigeria contributes significantly to this burden, with high incidence and mortality rates attributed to late presentation and inadequate preventive services [4,5].

Human papillomavirus (HPV) is a well-established necessary causal agent of cervical cancer [2,6]. About 15 known high-risk HPV genotypes cause approximately 95% of cervical cancer, while the low-risk HPV genotypes cause low-grade lesions such as genital warts [2,6,7]. The main route of transmission of HPV is sexual intercourse [6]. However, a few cases of vertical transmission of HPV have been reported [8]. Even though HPV infection can be cleared within 8-10 months, persistent infections occur when HPV evades the immune system, thus high-grade squamous intraepithelial lesions and cancers can occur [2,6,7,9].

Women living with human immunodeficiency virus (HIV) are at increased risk of HPV infection, persistence, and progression to cervical cancer due to immunosuppression [10,11]. Due to impaired cell-mediated immune response, HIV-positive women have reduced capacity to clear HPV and are at higher risk of developing cervical cancer [10]. Additionally, HIV has an indirect role in oncogenesis, mainly via immune suppression, enhancing the oncogenic effects of high-risk HPV [12]. Studies have consistently shown that HIV-positive women are more likely to acquire multiple HPV infections and harbor high-risk oncogenic genotypes compared to HIV-negative women [10]. A meta-analysis of 24 studies found that women living with HIV have a sixfold higher risk of developing cervical cancer relative to women without HIV [12].

Although HPV types 16 and 18 are responsible for approximately 70% of cervical cancer cases globally, regional variations in genotype distribution have been documented [13-16]. Emerging evidence suggests that other high-risk genotypes, such as HPV 52, 58, and 45, may play a more prominent role in certain populations, particularly among HIV-infected women [15]. The effectiveness of HPV vaccines in preventing the incidence of cervical cancer will be dependent on the prevalence of oncogenic vaccine genotypes in different populations [17].

Understanding the comparative prevalence and genotype distribution of HPV among HIV-positive and HIV-negative women is essential for informing targeted prevention strategies, including vaccination and screening programs. This study is novel because the molecular epidemiology approach with advanced genotyping was used to identify not just the presence of HPV infection, but specifically the genotypes as well as the multiple, co-occurring HPV strains, thus making the study provide more detailed information on viral diversity and distribution among HIV-positive and HIV-negative women in Lagos, Nigeria. Understanding the local distribution of HPV genotypes is critical to determine the effectiveness of HPV vaccines in preventing the incidence of cervical cancer among vulnerable groups in Nigeria.

The gap in the current screening approach may be inadequate, creating a need to know if targeted screening is required for HIV-positive individuals. The results of this study will justify the use of specific genotyping assays for HIV-positive women, potentially saving lives through earlier detection of fast-progressing cervical lesions. Ultimately, this study aims to provide the evidence base needed to reduce the disproportionate burden of cervical cancer mortality and close the disparity gap among HIV-positive and HIV-negative women in Nigeria.

This study was designed to address a critical public health disparity between these groups of women. The study will provide data to policymakers to allocate public health resources efficiently, focusing on the most prevalent cancer-causing HPV genotypes. This study, therefore, aimed to assess and compare the prevalence and genotype distribution of HPV infection among HIV-positive and HIV-negative women in Lagos University Teaching Hospital (LUTH) in Lagos State, Nigeria.

This research was part of a larger study that compared HPV DNA and Pap smear as cervical cancer screening tests among HIV-positive and HIV-negative women attending clinics at LUTH in Lagos State, Nigeria. The findings of cervical cancer screening using the Pap smear test among these two groups of women have been published [18]. This current article reports the findings of the comparison of cervical cancer screening using the HPV DNA test among HIV-positive and HIV-negative women.

## Materials and methods

Study area and site

Lagos State is the smallest state by landmass but the most populous in Nigeria. It is in southwest Nigeria with over 20 million residents [19]. Lagos State is Nigeria’s commercial capital; it is highly urbanized and densely populated, with a significant HIV burden [20]. There are 20 local government areas (LGAs) in Lagos State, with a mix of public and private health facilities [19]. This hospital-based study was conducted at LUTH, the teaching hospital of the College of Medicine, University of Lagos, located in Idi-Araba, Surulere, Lagos Mainland LGA. This tertiary health care facility provides specialized services, including HIV care and cervical cancer screening, such as HPV DNA screening, Pap smear, and cytopathology [21]. LUTH also collaborates with the AIDS Prevention Initiative in Nigeria (APIN), a non-governmental organization established in 2000 to support HIV/AIDS prevention, treatment, and research in Nigeria.

Study design and population

A comparative cross-sectional study design was used. The study population comprised asymptomatic HIV-positive women attending and receiving care and treatment at the APIN clinic of LUTH and HIV-negative women attending the General Outpatients Clinic at GOPD of LUTH in Lagos. HIV-positive and HIV-negative women aged 30-60 years (eligible age for cervical cancer screening) who provided informed consent were eligible for inclusion in this study. The HIV-positive group consisted of women with confirmed HIV infection, while the HIV-negative group included women with documented negative HIV status. Exclusion criteria included pregnancy, prior total hysterectomy, current treatment for cervical cancer, or refusal to participate. Women who were menstruating and those who had never had sexual intercourse were also excluded from this study.

Sample size determination

The minimum sample size was determined using the standard formula for comparing two independent proportions [22], based on the prevalence of HPV-positive results of 69.2% among HIV-positive women attending selected government hospitals in Ondo State, Nigeria [23]. An estimated difference of 15% in the prevalence of HPV-positive results between HIV-positive and HIV-negative women, a confidence interval of 95%, and a power of 90% were used in this calculation of the minimum sample size for this study. Allowing for a 20% non-response rate, a minimum sample size of 194 women per group was estimated.

Sampling technique

A systematic random sampling technique was employed to recruit study participants. Based on an average daily registration of 20 new women, approximately 800 women were registered over eight weeks at the APIN and General Outpatient (GOPD) clinics of LUTH. With an expected sample size of 200, a sampling interval (k) of 4 was established (Total Population [800]/Expected Study Participants [200] =4). To begin, a simple random method (balloting) was used to select the first participant from the first four eligible women registered. Subsequently, every fourth woman thereafter was selected for inclusion. If a selected participant did not meet the eligibility criteria, the next available eligible woman was recruited. This process maintained a daily target of five to eight participants to ensure sample representativeness over the two-month study period.

Data collection procedures

Sociodemographic and clinical data were obtained using a pretested, structured, and interviewer-administered questionnaire. Information collected included sociodemographic characteristics such as age and reproductive and sexual histories, as well as HIV and HPV status. HIV status was confirmed for all participants: test results for those in the APIN clinic were retrieved and documented, while participants in the GOPD clinic underwent rapid HIV screening conducted by a trained Voluntary Counselling and Testing (VCT) officer at the Outpatients Department of LUTH. Pre-test counseling was provided to all screened participants, and all GOPD clinic participants tested negative for HIV. 

Selected participants were invited to a private room designated for study within the two clinics, which was equipped with chairs, a table for specimen collection materials, and an examination couch with a privacy screen. After obtaining informed consent, trained research assistants administered the questionnaire. The researcher ensured all questionnaires were checked for completeness and consistency. 

Sample collection for the HPV genital sample was performed by trained clinicians, including an oncology gynecologist and a public health physician, assisted by a medical officer and a chaperone. Cervical samples were collected using a sterile cytobrush contained in a female sample collection kit manufactured by Hybribio Biochemical Company Limited. Following an explanation of the procedure and informed consent, cervical sample collection for HPV DNA testing was performed by the trained clinicians and assisted by a medical officer.

Participants were positioned in the dorsal lithotomy position. A sterile disposable bivalve speculum was gently inserted to visualize the cervix. The cervix and vaginal walls were inspected, and the squamocolumnar junction was identified. Cervical cells were collected by rotating the cytobrush 360° at the squamocolumnar junction. The collected specimens were immediately placed into the HPV DNA collection medium containing preservatives to maintain sample integrity. All samples were transported to the Department of Anatomic and Molecular Pathology, College of Medicine, University of Lagos, where they were stored at −20°C prior to analysis.

Laboratory analysis

HPV DNA testing was performed using validated molecular assays that detect multiple HPV genotypes simultaneously. HPV DNA testing and genotyping were performed using the HPV GenoArray Test Kit (Hybribio Biochemical Company Limited, China), which detects 21 HPV genotypes, including high-risk types (16, 18, 31, 33, 35, 39, 45, 51, 52, 56, 58, 59, 68), low-risk types (6, 11, 42, 43, 44, 81), and probable high-risk types (53 and 66). The assay utilizes polymerase chain reaction (PCR) combined with flow-through hybridization for qualitative detection and genotyping.

DNA extraction was performed by cell lysis and centrifugation. Amplification was carried out using a thermal cycler under standard conditions, followed by hybridization on a membrane containing genotype-specific probes. Detection was based on a colorimetric reaction indicating the presence of specific HPV DNA types.

All assays were conducted by trained laboratory scientists and supervised by a consultant pathologist, and they followed the manufacturer’s protocol. Quality control measures included the use of positive and negative controls in each batch to ensure assay validity and prevent contamination.

HPV results were provided as identified genotypes and were classified into high-risk (oncogenic) and low-risk categories based on established classifications by the International Agency for Research on Cancer. The HPV results were documented and validated by the supervising pathologist. The participants were contacted by telephone or at their clinic appointment dates, and they were notified of their results. Participants who had HPV DNA-negative test results were assured and informed to repeat the test after five years. Participants with positive high-risk HPV DNA test results were referred immediately to the Gynecological Clinic at LUTH for further assessment and treatment.

Outcome measures

The primary outcomes that were compared across HIV status were the prevalence of any HPV infection, distribution of high-risk and low-risk HPV genotypes, and frequency of single and multiple HPV infections.

Secondary outcomes included genotype-specific prevalence and comparison across HIV status.

Statistical analysis

Data were analyzed using IBM Corp. Released 2020. IBM SPSS Statistics for Windows, Version 26. Armonk, NY: IBM Corp. Descriptive statistics were computed for all variables. Categorical variables were summarized using frequencies and percentages, while continuous variables (if applicable) were summarized using means and standard deviations.

Bivariate analysis was conducted to assess associations between HIV status and HPV infection outcomes. The Pearson chi-square (χ²) test was used to compare proportions between HIV-positive and HIV-negative groups. Where expected cell counts were less than five, Fisher’s exact test was applied to ensure the validity of statistical inference. The likelihood ratio chi-square was used where appropriate. Statistical significance was set at p < 0.05.

Ethical considerations

Ethical approval for the larger study was obtained from the College of Medicine, University of Lagos, Research and Ethics Committee on the 2nd of November 2021, with reference number CMUL/HREC/09/21/921 [18]. Permission was sought and obtained from the heads of the clinics (APIN and GOP Clinics) before the commencement of this study. Written informed consent was obtained from all participants before enrolment. Participation was voluntary, and confidentiality of participant information was strictly maintained throughout the study. All the study participants received health education on cervical cancer and the preventive methods, and they were all given contact information to call for further assistance. Participants who were positive for high-risk HPV DNA tests were referred to the Gynecology Unit of LUTH for further evaluation and treatment. All the procedures were in accordance with the ethical standards of the committee responsible on human experimentation (institutional and national) and adhered to the ethical principles outlined in the Helsinki Declaration of 1975, as revised in 2000.

## Results

A total of 441 women participated in the study, comprising 232 HIV-positive and 209 HIV-negative women. The mean age ± standard deviation (SD) of the respondents was 42.47 ± 8.05 years. HIV-positive women were slightly older compared to HIV-negative women, with the mean age of 42.63 ± 7.44 years and 42.30 ± 8.68 years, respectively.

The prevalence of HPV infection using the HPV DNA test among HIV-positive and HIV-negative women is shown in Table 1. The overall prevalence of human papillomavirus (HPV) infection was 38.8% (171/441). The prevalence of HPV infection was significantly higher among HIV-positive women (50.4% [117/232]) compared to HIV-negative women (25.8% [54/209]) (χ² = 28.013, p < 0.0001). Among those infected, high-risk HPV genotypes predominated in both groups but were significantly more frequent among HIV-positive women (91.5% [107/117]) than HIV-negative women (68.5% [37/54]) (χ² = 32.168, p < 0.0001). Conversely, low-risk HPV infections were less common among HIV-positive women (8.5% [10/117]) compared to HIV-negative women (20.4% [11/54]). Mixed infections (high- and low-risk genotypes) were observed only among HIV-negative women (11.1% [6/54]).

**Table 1:** Comparison of the prevalence of HPV infection among the respondents by HIV status † Statistically significant, ‡ Likelihood ratio Data were presented as n (%), and the p-value was considered significant at p<0.05.

Variable	HIV positive	HIV negative	Total	Chi square	p-value
	n=232	n=209	n=441	ꭓ^2^	
	Freq. (%)	Freq. (%)	Freq. (%)		
HPV infection					
No HPV infection	115 (49.6)	155 (74.2)	270 (61.2)	28.013	<0.0001 †
Any HPV infection	117 (50.4)	54 (25.8)	171 (38.8)		
HPV infection					
No HPV infection	115 (49.6)	155 (74.2)	270 (61.2)	32.168	<0.0001 †
Low-risk HPV infection	10 (4.3)	11 (5.3)	21 (4.8)		
High-risk HPV infection	107 (46.1)	43 (20.6)	150 (34.0)		
Specific HPV genotypes	n = 117	n = 54	n = 171		
Low-risk HPV infection	10 (8.5)	11 (20.4)	21 (12.3)	20.112	<0.0001 ‡†
High-risk HPV infection	107 (91.5)	37 (68.5)	144 (84.2)		
Mixed - high and low-risk HPV	0 (0.0)	6 (11.1)	6 (3.5)		
Number of HPV infections	n = 117	n = 54	n = 171		
Single HPV infection	56 (47.9)	34 (63.0)	90 (52.6)	3.379	0.066
Multiple HPV infections	61 (52.1)	20 (37.0)	81 (47.4)		
Multiple HPV infection	n = 61	n = 20	n = 81		
Double HPV genotypes	34 (55.7)	10 (50.0)	44 (54.3)	1.665	0.645 ‡
Triple HPV genotypes	13 (21.3)	7 (35.0)	20 (24.7)		
Quadruple HPV genotypes	4 (6.6)	1 (5.0)	5 (6.2)		
More than 5 HPV genotypes	10 (16.4)	2 (10.0)	12 (14.8)		

Multiple HPV infections were slightly more common among HIV-positive women (52.1% [61/117]) compared to HIV-negative women (37.0% [20/54]), although this difference was not statistically significant (χ² = 3.379, p = 0.066). Among women with multiple infections, double HPV genotypes were the most frequent in both groups.

The prevalence of HPV DNA genotypes among HIV-positive and HIV-negative women is shown in Table 2. Analysis of genotype distribution revealed 15 high-risk HPV types across both groups. HIV-positive women had a significantly higher prevalence of several oncogenic genotypes, including HPV 18 (8.6% [20/232] vs. 2.9% [6/209], p = 0.010), HPV 35 (5.2% [12/232] vs. 1.4% [3/209], p = 0.036), HPV 45 (8.2% [19/232] vs. 1.4% [3/209], p = 0.002), HPV 52 (12.1% [28/232] vs. 4.3% [9/209], p = 0.003), HPV 53 (9.5% [22/232] vs. 2.9% [6/209], p = 0.004), HPV 58 (9.1% [21/232] vs. 3.3% [7/209], p = 0.014), and HPV 68 (6.0% [14/232] vs. 1.0% [2/209], p = 0.004).

**Table 2:** Comparison of the prevalence of HPV DNA genotypes among the respondents by HIV status † Statistically significant, § Fisher’s Exact test Data were presented as n (%), and the p-value was considered significant at p<0.05.

Genotypes	HIV positive	HIV negative	Total	Chi Square	p-value
	n=232	n=209	n=441	ꭓ^2^	
	Freq. (%)	Freq. (%)	Freq. (%)		
High-risk genotypes					
HPV 16	11 (4.7)	6 (2.9)	17 (3.9)	1.038	0.308
HPV 18	20 (8.6)	6 (2.9)	26 (5.9)	6.552	0.010 †
HPV 31	12 (5.2)	7 (3.3)	19 (4.3)	0.886	0.346
HPV 33	9 (3.9)	2 (1.0)	11 (2.5)	3.861	0.066 §
HPV 35	12 (5.2)	3 (1.4)	15 (3.4)	4.673	0.036 §†
HPV 39	6 (2.6)	6 (2.9)	12 (2.7)	0.034	0.854
HPV 45	19 (8.2)	3 (1.4)	22 (5.0)	10.583	0.002 §†
HPV 51	7 (3.0)	5 (2.4)	12 (2.7)	0.162	0.687
HPV 52	28 (12.1)	9 (4.3)	37 (8.4)	8.620	0.003 †
HPV 53	22 (9.5)	6 (2.9)	28 (6.3)	8.084	0.004 †
HPV 56	4 (1.7)	2 (1.0)	6 (1.4)	0.482	0.688 §
HPV 58	21 (9.1)	7 (3.3)	28 (6.3)	6.013	0.014 †
HPV 59	5 (2.2)	2 (1.0)	7 (1.6)	1.011	0.454 §
HPV 66	8 (3.4)	2 (1.0)	10 (2.3)	3.079	0.110 §
HPV 68	14 (6.0)	2 (1.0)	16 (3.6)	8.107	0.004 §†
Low-risk genotypes					
HPV 6	8 (3.4)	8 (3.8)	16 (3.6)	0.045	0.831
HPV 11	3 (1.3)	2 (1.0)	5 (1.1)	0.110	1.000 §
HPV 42	6 (2.6)	0 (0.0)	6 (1.4)	5.480	0.031 §†
HPV 43	1 (0.4)	1 (0.5)	2 (0.5)	0.005	1.000 §
HPV 44	14 (6.0)	2 (1.0)	16 (3.6)	8.107	0.004 §†
HPV 81	15 (6.5)	7 (3.3)	22 (5.0)	2.253	1.133

Six low-risk HPV genotypes were detected among HIV-positive women and five among HIV-negative women. Significant differences were observed for HPV 42 (2.6% [6/232] vs. 0.0% [0/209], p = 0.031) and HPV 44 (6.0% [14/232] vs. 1.0% [2/209], p = 0.004), both of which were higher in HIV-positive women.

Overall, HPV 52 was the most prevalent high-risk genotype (8.4% [37/441]), while HPV 81 was the most common low-risk genotype (5.0% [22/441]) in the study population.

**Figure 1:**
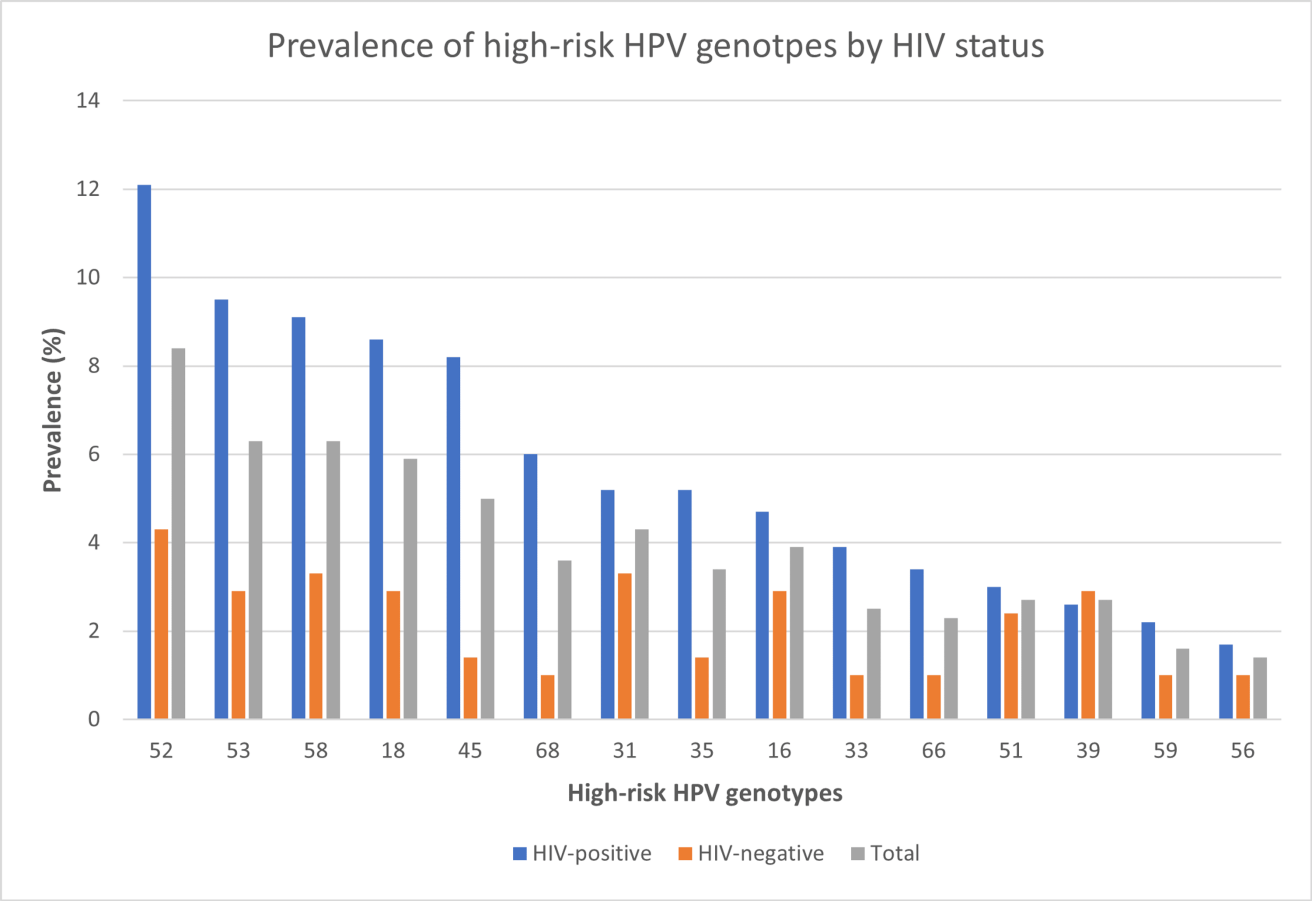
Comparison of the prevalence and distribution of high-risk HPV genotypes among the respondents by HIV status

Figure 1 shows the prevalence and distribution of high-risk HPV genotypes among the HIV-positive and HIV-negative women in the most prevalent order. The most prevalent high-risk HPV genotype in the study population was HPV 52 [8.4% (37/441)]. Others were HPV53, HPV58, HPV18, HPV45, HPV68, HPV31, HPV35, HPV16, HPV33, HPV66, HPV51, HPV39, HPV59, and HPV56 in descending order.

**Figure 2:**
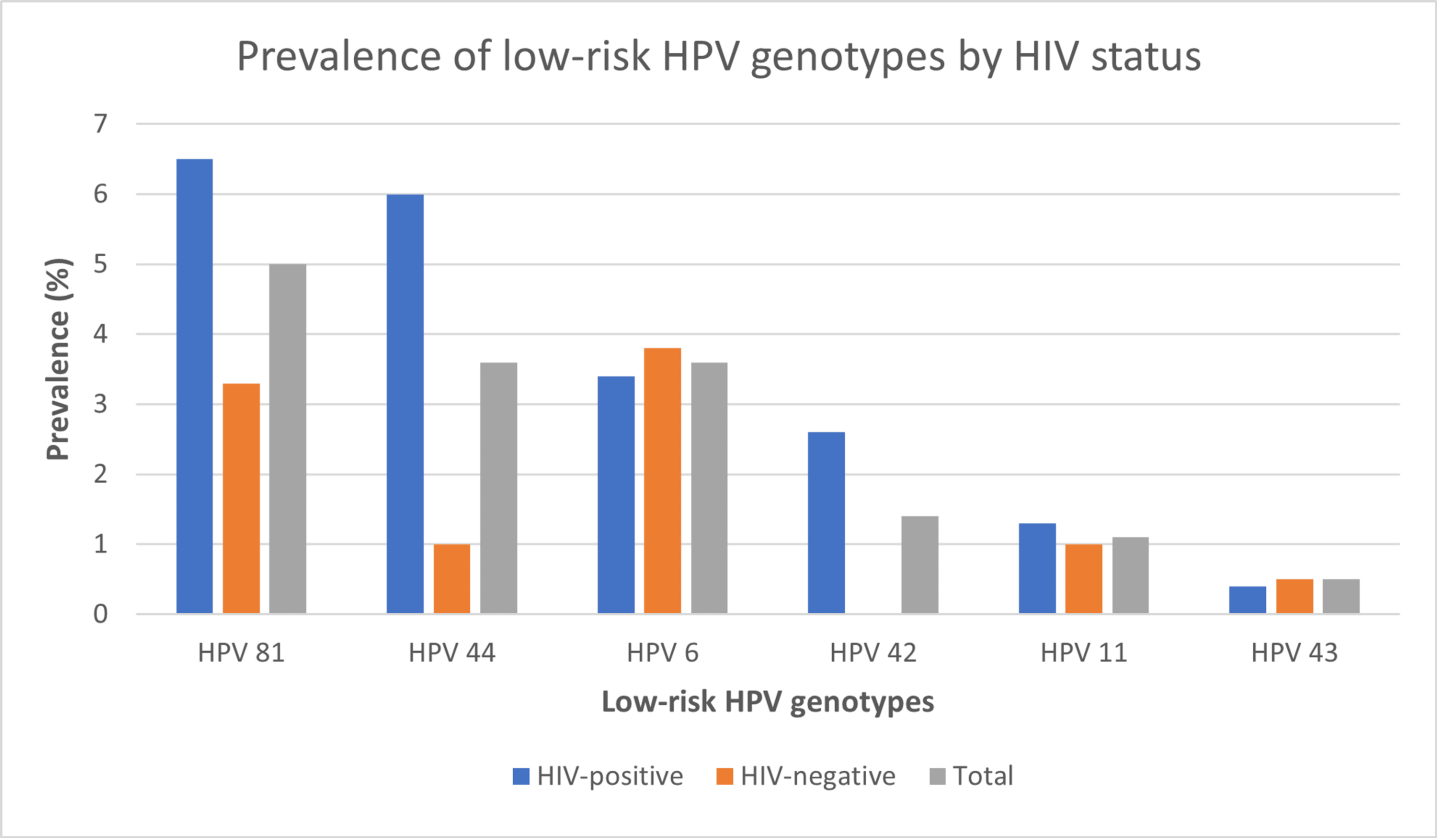
Comparison of the prevalence and distribution of low-risk HPV genotypes among the respondents by HIV status

Figure 2 shows the prevalence and distribution of low-risk HPV genotypes among the HIV-positive and HIV-negative women in the most prevalent order. The most prevalent low-risk HPV genotype in the study population was HPV 81 (5% [22/441]). Others were HPV44, HPV6, HPV42, HPV11, and HPV43 in descending order.

## Discussion

This study demonstrates a significantly higher prevalence of HPV infection among HIV-positive women compared to HIV-negative counterparts, corroborating existing evidence that immunosuppression associated with HIV increases susceptibility to persistent HPV infection [10,15]. The observed prevalence among HIV-positive compared to HIV-negative women (50.4% vs. 25.8%) aligns with prior epidemiological findings indicating a two- to threefold higher burden of HPV among HIV-infected populations [10,24].

The predominance of high-risk HPV genotypes among HIV-positive women is of clinical relevance. The significantly higher proportion of high-risk oncogenic HPV types (91.5%) suggests an elevated risk of progression to cervical intraepithelial neoplasia and invasive cervical cancer in this group [2,6]. This supports the biological plausibility that impaired immune surveillance in HIV infection facilitates persistence and oncogenic transformation of HPV [11].

The absence of mixed HPV infections (high-risk and low-risk HPV infections) among HIV-positive women, contrasted with their presence in HIV-negative women, is noteworthy and may reflect differences in viral competition, immune modulation, or detection sensitivity [25]. However, the higher prevalence of multiple HPV infections among HIV-positive women, although not statistically significant, is consistent with the literature suggesting that immunocompromised individuals are more likely to harbor multiple concurrent infections [11]. The genotype-specific analysis revealed a higher burden of several high-risk HPV types (notably HPV 18, 45, 52, 53, 58, and 68) among HIV-positive women [13,15]. Of note, HPV 52 emerged as the most prevalent genotype overall, diverging from the global predominance of HPV 16 and 18. Studies in other areas, such as Black rural women in Southeastern Brazil and women with cervical lesions in China, have also shown a higher frequency of high-risk HPV 52 compared to HPV 16 [26,27]. These findings underscore regional variation in HPV genotype distribution and have implications for vaccine effectiveness, particularly in settings where non-16/18 genotypes contribute substantially to disease burden [28].

Therefore, current knowledge of the distribution of HPV subtypes in each region still requires further verification through large-scale studies, as this may significantly impact the formulation of strategies for screening, treatment, prevention, and vaccine development for HPV-attributable diseases. The HPV vaccine available in Nigeria is primarily the single-dose quadrivalent HPV vaccine, specifically Gardasil-4 (protects against four types of HPV: 6, 11, 16, and 18), introduced by the Federal Government in 2023 into its national routine immunization schedule, which is available for girls aged 9-14 years at public primary health care centers free of charge [29,30]. Other HPV vaccines, such as bivalent Cervarix (protects against the two highest-risk types of HPV: 16 and 18) or nonavalent Gardasil-9 (protects against nine types of HPV: 6, 11, 16, 18, 31, 33, 45, 52, and 58), are available for a fee at private clinics.

Understanding the differences in the epidemiology of high-risk HPV infection is vital, given that the current HPV vaccines may not include some of the types that are prevalent among HIV-positive and HIV-negative women in Nigeria. This will be particularly important if there is little or no antibody cross-reactivity between current vaccines and the high-risk HPV genotypes prevalent in Nigeria. With the high prevalence of non-16 and non-18 high-risk HPV genotypes among HIV-positive women in Nigeria, the current HPV vaccine in the national routine immunization schedule may have a limited impact on this group of the population [17].

The higher prevalence of certain low-risk genotypes among HIV-positive women further reflects increased susceptibility to HPV infection generally, although these types are less clinically significant in oncogenesis. Overall, these findings highlight the synergistic interaction between HIV and HPV infections and reinforce the need for targeted cervical cancer prevention strategies among HIV-positive women.

Limitations of the study

This study should be interpreted considering some methodological limitations. First, the cross-sectional design precludes causal inference; while an association between HIV infection and increased HPV prevalence was observed, temporal relationships and progression dynamics cannot be established. Second, the study may be subject to selection bias, as participants were recruited from healthcare settings, potentially limiting generalizability to the broader population of women, particularly those with known and unknown HIV status who had limited access to care. Third, although the sample size may be adequate for primary comparisons, it may have limited the statistical power to detect significant differences in subgroup analyses, such as multiple HPV infections or genotype-specific distributions. Fourth, reliance on a single time point HPV DNA assessment may underestimate transient infections and does not capture persistence, which is the key determinant of progression to cervical neoplasia. In addition, potential confounding variables, such as sexual behavior, antiretroviral therapy adherence, duration of HIV infection, immune status (e.g., CD4 count), and co-existing sexually transmitted infections, were not controlled for, which may influence both HPV acquisition and persistence. Finally, the absence of detailed immunological and virological markers limits deeper mechanistic interpretation of the observed associations.

Implications for policy

The findings underscore the need for strengthened integration of cervical cancer prevention services within HIV care programs. Policymakers should prioritize routine HPV screening and cervical cancer screening for HIV-positive women as part of comprehensive HIV management. Given the high prevalence of non-16/18 high-risk HPV genotypes, national immunization strategies may need to consider broader-spectrum vaccines and support ongoing surveillance of genotype distribution to inform vaccine policy.

Furthermore, resource allocation should emphasize scaling up access to high-performance HPV testing, particularly in high-burden settings. Policies that promote equitable access to screening, early detection, and treatment services for vulnerable populations, including women living with HIV, are critical to reducing cervical cancer incidence and mortality.

Implications for practice

Clinically, the elevated burden of high-risk HPV among HIV-positive women highlights the necessity for more intensive and tailored screening protocols, including earlier initiation and more frequent screening intervals. Healthcare providers should maintain a high index of suspicion for cervical precancerous lesions in this population and ensure timely referral and management.

The findings also support the integration of HPV vaccination into HIV care services, even among adult women, where appropriate. In addition, health education interventions should be strengthened to improve awareness of HPV-related risks and promote uptake of preventive services. Multidisciplinary approaches involving gynecologists, infectious disease specialists, and primary care providers are essential to optimize outcomes.

Implications for research

Future research should employ longitudinal designs to better elucidate the natural history of HPV infection among HIV-positive women, particularly the persistence and progression of non-16/18 high-risk genotypes. Large-scale, multicenter studies are needed to validate regional genotype patterns and enhance the external validity of findings.

Further investigation into immunological correlates, including the role of immune suppression and antiretroviral therapy in HPV persistence and clearance, is needed. Additionally, studies evaluating the effectiveness of existing HPV vaccines against prevalent non-16/18 genotypes in African populations are essential.

Operational research should also explore cost-effective screening strategies and implementation models suitable for low-resource settings, as well as interventions aimed at improving screening uptake and adherence to follow-up care.

## Conclusions

The study demonstrates a significantly higher prevalence of HPV infection, particularly high-risk oncogenic genotypes, among HIV-positive women compared to HIV-negative women. HIV infection is strongly associated with increased susceptibility to HPV infection and a higher burden of carcinogenic HPV types. The predominance of non-HPV 16/18 genotypes, especially HPV 52, indicates important regional epidemiological patterns with implications for prevention strategies.

There should be enhanced screening programs for HIV-positive women. They should be prioritized for regular and more frequent cervical cancer screening using HPV DNA testing, given their higher risk of persistent and high-risk HPV infections. Cervical cancer screening should be fully integrated into HIV care and treatment programs to ensure early detection and management of HPV-related lesions. The vaccination policy should be reviewed; the national immunization programs should consider the inclusion and wider use of polyvalent HPV vaccines (e.g., nonavalent vaccines) that cover additional high-risk genotypes such as HPV 52 and 58, which are prevalent in this population. Targeted public health education campaigns should be implemented to raise awareness about HPV infection risks, especially among women living with HIV. Longitudinal studies are recommended to assess the persistence, progression, and clinical outcomes of HPV infections among HIV-positive women, as well as vaccine effectiveness in this subgroup. Policymakers should develop context-specific cervical cancer prevention guidelines that account for the higher HPV burden among HIV-infected populations.
